# Countering the negative effects of dietary soybean (SB) meal in Nile tilapia with organic acid salts

**DOI:** 10.1038/s41598-026-45042-x

**Published:** 2026-04-17

**Authors:** Abeer Awad, Hanan A. Ghetas, Mohamed A. Khallaf, Mustafa Shukry, Ali A. Soliman, Mahmoud S. Gewaily, Asem A. Amer, Akram Ismael Shehata, Hany M. R. Abdel-Latif

**Affiliations:** 1 Department of Aquatic Animal Medicine and Management, Faculty of Veterinary Medicine, University of Sadat City, Sadat City, Egypt; 2 Department of Physiology, Faculty of Veterinary Medicine, Kafrelsheikh University, Kafrelsheikh, 33516 Egypt; 3 Department of Biomedical Sciences, College of Veterinary Medicine, King Faisal University, P.O. Box 400, 31982 Saudi Arabia, Al-Ahsa Saudi Arabia; 4 National Institute of Oceanography and Fisheries (NIOF), Cairo, Egypt; 5 Department of Anatomy and Embryology, Faculty of Veterinary Medicine, Kafrelsheikh University, Kafrelsheikh, 33516 Egypt; 6 Department of Fish Nutrition, Central Laboratory for Aquaculture Research, Sakha Aquaculture Research Unit, Agriculture Research Center, Abbassa, Sharkia Egypt; 7 Department of Animal and Fish Production, Faculty of Agriculture (Saba Basha), Alexandria University, Alexandria, 21531 Egypt; 8 Institute of Marine Sciences, Shantou University, Shantou, 515063 China; 9 Department of Poultry and Fish Diseases, Faculty of Veterinary Medicine, Alexandria University, Alexandria, 22758 Egypt

**Keywords:** Digestive enzyme, Nile tilapia, Organic acid, Soybean meal, Enteritis, Biochemistry, Biotechnology, Immunology, Physiology, Zoology

## Abstract

While soybean meal (SBM) is a cost-effective protein source for Nile tilapia, its anti-nutritional factors require dietary interventions such as the supplementation of organic acid salts to maintain health and gut functions. Therefore, this study evaluated the dietary effects of organic acid salts as sodium propionate (PR), sodium butyrate (BT), and their mixture (PR + BT) on the growth, digestive enzymes, hepatic antioxidant responses and intestinal histomorphology of Nile tilapia fed a high SBM diet. Fingerlings (average initial weight ~ 19.65 ± 0.1 g) were assigned to five triplicate diets for 12 weeks: a negative control (CNT, contains 350 g/kg SBM), a high SBM diet (SB, contains 550 g/kg SBM), SB + PR (2.0 g/kg), SB + BT (6.25 g/kg), and their mixture (SB + PR + BT). Results revealed that fish fed solely on SB diet had significantly reduced growth, feed efficiency, and digestive enzyme activities, alongside higher levels of oxidative stress markers (MDA malondialdehyde levels) and intestinal inflammatory lesions (enteritis). Supplementation with PR, BT, or their mixture improved final body weight, weight gain, and feed conversion ratio. Digestive enzyme activities (lipase, amylase, protease) and antioxidant defenses (T-AOC, SOD, CAT, and GPx) were improved, while lipid peroxidation (MDA) was reduced in the treated groups. The histological examination confirmed that organic acid salts supplementation, especially PR + BT, alleviated SB-induced enteritis. Gene expression analysis revealed enhanced growth (*IGF-2*, *GHR1*, *IGF-1*, and *GHR2*) and immune-related markers (*TLR2*, and *IFN-γ*) and suppression of inflammatory (*IL-1β*, *TNF-α*, and *caspase-3*) related genes in the treated groups. In conclusion, PR and BT supplementation, especially in combination, effectively counteracted the negative impacts of high SB diets, supporting growth, alleviating inflammation, mitigating oxidative stress, and improving intestinal health of fish. These effects underscore the fascinating roles of organic acid salts in the improvement of aquafeed for tilapia culture.

## Introduction

Tilapia aquaculture has witnessed a remarkable global expansion over the past three decades, establishing itself as one of the most important sectors of aquaculture worldwide^1,2^. The intensification of tilapia farming systems, however, has necessitated increased reliance on various feed additives to sustain growth, maintain health, and enhance water quality^3–5^. These additives include herbal extracts, probiotics, prebiotics, organic acids (OAs), exogenous enzymes^6,7^, and several others. However, growing concerns regarding antibiotic resistance and residue accumulation have driven a shift toward functional feed additives, which are regarded as safer alternatives for stimulating host immunity and reducing disease incidence in cultured fish^8–11^.

OAs, defined as oxygenated compounds with one or more carboxylic groups, are categorized into short-chain fatty acids (e.g., acetic, citric, formic, propionic, and lactic acids) and medium-chain fatty acids (e.g., lauric, caprylic, and capric acids)^12–15^. These compounds, and their salts (typically sodium, potassium, or calcium salts), are classified as GRAS “generally regarded as safe” compounds and have been widely employed in livestock as growth promoters^11,16^. Their application in aquaculture has expanded considerably in recent years, with numerous reports indicating improvements in growth, nutrient utilization, mineral bioavailability, immune competence, and intestinal microbiota balance in fish and crustaceans^13,17,18^. Mechanistically, OAs exert multiple beneficial effects: they lower digesta pH, inhibit pathogenic bacteria by disrupting cytoplasmic pH, enhance pancreatic enzyme secretion, improve gut morphology, and act as readily available energy substrates^19–21^.

Sodium butyrate and sodium propionate are short-chain fatty acids widely recognized for their roles in enhancing gut health, modulating immunity, and improving growth in fish nutrition strategies^22^. Despite these promising attributes, the responses of aquatic animals to dietary OAs remain inconsistent and also species-specific. For instance, sodium propionate suppressed growth in Arctic char (*Salvelinus alpinus*)^23^, but enhanced performance in zebrafish (*Danio rerio*)^24^, common carp (*Cyprinus carpio*)^25^, and Caspian white fish (*Rutilus kutum*)^26^. Similarly, sodium diformate improved mineral digestibility in rainbow trout (*Oncorhynchus mykiss*)^27^, while a blend of sodium formate and butyrate negatively affected nutrient utilization in the same species^28^. In tilapia, dietary sodium citrate (1–4%) proved toxic, impairing growth and causing liver necrosis and hemorrhage^29^, whereas citric acid has been reported to improve growth and feed efficiency in other species^30,31^. These contrasting outcomes underscore the complexity of OAs and host interactions and highlight the necessity of several species-specific investigations.

Beyond growth, recent research has revealed important immunomodulatory and gut health-promoting effects of dietary OAs. For example, malic acid supplementation enhanced lysozyme and antioxidant activities in rainbow trout without altering growth performance^32^, while butyric acid improved intestinal villi height in giant grouper (*Epinephelus lanceolatus*)^33^. Similarly, dietary formic acid enhanced hematological and biochemical indices and reduced mortality in Nile tilapia challenged with *Aeromonas veronii*^34^.

A major challenge in tilapia nutrition is the replacement of fishmeal with soybean meal (SB), an economical and sustainable protein source that is increasingly adopted in commercial aquafeeds^35,36^. However, high dietary levels of SB are associated with intestinal inflammation, altered gut morphology, impaired nutrient absorption, and reduced performance in tilapia and other fish species^37,38^. In this context, dietary strategies that mitigate the negative consequences of SB-based diets are urgently needed. Functional additives such as OAs hold promise, given their capacity to enhance intestinal histoarchitecture, suppress SB-induced enteritis, and restore microbial homeostasis^39,40^. While probiotics and phytobiotics have been extensively studied in SB-based tilapia diets^41,42^, the roles of OAs in this context remain poorly understood. Therefore, the novelty of the present study lies in elucidating the functional roles of sodium propionate, sodium butyrate, and their mixture in ameliorating the adverse effects of high SB inclusion in Nile tilapia diets. Few studies have specifically addressed their potential in counteracting SB-induced enteropathy and enhancing intestinal health in tilapia. Given the economic importance of SB and the global reliance on tilapia as a farmed species, this investigation provides critical insights into the development of sustainable feeding strategies that improve gut health and immunity.

## Materials and methods

### Pre-trial conditioning of fish and Ethical approval

Nile tilapia (*Oreochromis niloticus*) fingerlings were obtained from a private farm in Kafr Elsheikh City, Egypt, and maintained in five 500 L fiberglass tanks with continuous aeration. Prior to the feeding trial, fish were acclimated for 2 weeks under controlled conditions and offered a balanced commercial diet (Aller Aqua Co., Egypt) to apparent satiation. During acclimation, water temperature (26–28 °C), dissolved oxygen (> 6.5 mg/L), and pH (7.6–7.9) were maintained within the optimal range for tilapia rearing. All experimental procedures were reviewed and approved by the Local Experimental Animal Care Committee, Faculty of Veterinary Medicine, Alexandria University, Egypt (Approval No. AU-013/2023/03/11-3R/4P/228). Additionally, all study techniques adhered to the ARRIVE criteria, version 2.030, ensuring that the study procedures followed accepted ethical guidelines and safeguarded the welfare of the fish subjects.

### Formulation of experimental diets with organic acid salts

Sodium propionate (CAS No.: 137-40-6) and sodium butyrate (CAS No.: 156-54-7) were commercially purchased from West Bengal Chemical Industries Limited, West Bengal, India. Five experimental diets were prepared and defined as a negative control (CNT, contains 350 g/kg SB meal), a high SB meal (SB, 550 g/kg soybean meal), and three treatment diets contains high SB and supplemented with sodium propionate (SB + PR, 2.0 g/kg), sodium butyrate (SB + BT, 6.25 g/kg), or their combination (SB + PR + BT). Sodium propionate dietary dose was selected according to the recommendation of Ding, et al.^43^, while the dietary application dose of sodium butyrate was selected according to the recommendation of Abd El-Naby, et al.^44^. All ingredients were precisely weighed, ground, and thoroughly mixed to ensure homogeneity. OAs were incorporated during mixing to achieve uniform distribution. The diets were then pelletized using a laboratory feed pelletizer at a 2.0 mm pellet size and then left to be dried in the air for 12 h. The formulated dried diets were then stored at 4 °C in airtight containers until use. All diets were formulated to meet the nutritional requirements of Nile tilapia according to NRC^45^ recommendations (Table 1).

**Table 1:** Feed ingredients (g) and proximate chemical composition (% on a dry weight basis) of the test diets.

Feed ingredients	Experimental diets				
	Diet 1	Diet 2	Diet 3	Diet 4	Diet 5
Fish meal	70	70	70	70	70
Soybean meal (SB)	350	550	550	550	550
Corn gluten (63%)	120	0	0	0	0
Ground corn (8.3%)	195	215	215	215	215
Rice bran	179	79	79	79	79
Fish oil	20	20	20	20	20
Starch	35	35	32.5	28.75	26.25
Di-calcium phosphate ^a^	10	10	10	10	10
Mineral premix ^a^	10	10	10	10	10
Vitamin premix ^a^	10	10	10	10	10
Vitamin C ^a^	0.5	0.5	0.5	0.5	0.5
Antioxidant ^b^	0.5	0.5	0.5	0.5	0.5
Sodium propionate (Na PR) ^c^	-	-	2.5	-	2.5
Sodium butyrate (Na BT) ^c^	-	-	-	6.25	6.25
Total (g)	1000	1000	1000	1000	1000
Proximate chemical analysis (% on a dry weight basis)					
Dry matter	89.46	89.0	89.0	88.45	88.46
Crude protein (CP)	31.23	31.09	31.09	31.09	31.09
Crude fiber (CF)	4.60	4.96	4.95	4.95	4.95
Ether extract (EE)	6.44	6.10	6.10	6.10	6.10
Ash	5.75	6.24	6.24	6.24	6.24
Nitrogen-free extract (NFE) ^d^	51.98	51.61	51.61	51.62	51.63
Gross energy (GE; Kcal/100 g) ^e^	467.386	463.371	463.372	463.374	463.376

^a^These ingredients were commercially purchased from AGRI-VET company for Manufacturing Vitamins and Feed Additives (Cairo, 10th of Ramadan City A2, Egypt).

^b^BHT (Butylated Hydroxytoluene) was used as an antioxidant.

^c^Sodium propionate (Na PR) and sodium butyrate (Na BT) were commercially purchased from West Bengal Chemical Industries Limited, West Bengal, India.

^d^NFE was calculated as 100 − (CP + EE + CF + Ash).

^e^GE was calculated = [(5.6 × CP) + (9.4 × EE) + (4.1 × (CF + NFE)], based on the values of values for proteins, lipids, and carbohydrates as 23.7, 38.7, and 16.9 kJ/g respectively.

### Experimental design, fish grouping, and feeding management

After a 2-week acclimation period, fingerlings (average weight ~ 19.65 ± 0.1 g) were randomly assigned to five experimental groups, each consisting of three replicates (Table 2). Fish were stocked at 10 individuals per hapa with dimensions (0.7 m × 0.7 m × 1.0 m). Twelve (12) hapas suspended within cement raceway ponds at the Baltim Research Station, National Institute of Oceanography and Fisheries, Egypt were used to carry out this experiment. The feeding trial continued for 12 weeks. Fish were fed their respective experimental diets twice daily (at 8:00 a.m. and 5:00 p.m.) with a daily feeding rate of 3% of the wet body weight. Feed quantities were adjusted bi-monthly, and any uneaten feed and feces were removed daily to maintain optimal water quality. To ensure optimal rearing conditions, approximately one-third (1/3) of the water was replaced daily with fresh water, and continuous aeration was provided in all hapas. A 12-h light and 12-h dark cycle was maintained throughout the study. Key water quality parameters, including temperature (26.7–28.4 °C), dissolved oxygen (6.70 ± 0.37 mg/L), pH (7.75–7.9), and un-ionized ammonia (0.02–0.22 mg/L), were monitored and consistently maintained within the optimal range for tilapia culture.

**Table 2:** Experimental design showing the distribution of treatments.

Experimental groups	Soybean Meal (SB, g/kg)	Supplement(s)	Supplement Level (g/kg diet)
CNT (Negative Control)	350	None	–
SB (Positive Control) (High soya bean meal)	550	None	–
SB + PR	550	Sodium propionate (Na PR)	2.0
SB + BT	550	Sodium butyrate (Na BT)	6.25
SB + PR + BT	550	A mixture of Na PR and Na BT	2.0 + 6.25

### Assessment of growth performance and survival

At the end of the feeding trial, fish were fasted for 24 h to standardize digestive status before sampling. Subsequently, fish were gently anesthetized with clove oil (20 mg/L) to minimize stress during handling. Individuals from each replicate hapa were carefully counted and weighed to assess growth, feed utilization, and survival. The key performance indicators were calculated to capture the growth response and efficiency of the diets:

Weight gain (WG; g) = Final weight (W_2_; g) − Initial weight (W_1_; g).

Specific growth rate (SGR, %/day)

$$\mathrm{SGR}=\frac{\text{Ln }{\mathrm{W}}_{2}-\text{Ln }{\mathrm{W}}_{1}}{\text{Feeding } \mathrm{period}}\times 100$$

Feed intake (FI, g)

$$FI, g /Fish =\frac{Total consumed feed, g}{Number of Fish}$$

Feed conversion ratio (FCR)

$$\mathrm{FCR}=\frac{\mathrm{Total} \text{ feed} \text{ intake} (\mathrm{g})}{\mathrm{WG} (\mathrm{g})}$$

Survival rate (SR%)

$$\text{SR \%}=\frac{\text{Number of surviving fish}}{\text{Initial } \text{number } \text{of } \mathrm{fish}}\times 100$$

### Profiling of intestinal digestive enzyme activities

The activities of intestinal digestive enzymes (α-amylase, lipase, and protease) were determined in the mid-intestinal region using commercially available colorimetric assay kits (Spectrum Diagnostic Co., Egypt), in accordance with previously described methodologies^46–48^. For this purpose, about 100 mg of intestinal tissue was homogenized in phosphate-buffered saline at a ratio of 1:9 (w/v). The tissue homogenates were centrifuged at 600×*g* for 10 min at 4 °C, after which the resulting supernatant was collected and stored at − 80 °C until further examination. Enzymatic activities were standardized and reported as units per milligram of protein (U/mg protein).

### Histological examination of intestines

Twelve (12) fish were randomly selected from each treatment group, anesthetized, and aseptically dissected. Intestinal samples were then collected from three distinct segments (the foregut, midgut, and hindgut) for each individual. Tissue fragments of approximately 0.5 cm^3^ were excised and preserved in 10% neutral buffered formalin for 24 h. Following fixation, samples were subjected to graded ethanol dehydration, cleared in xylene, and subsequently embedded in paraffin wax. Serial sections of 5 μm thickness were prepared using a rotary microtome. For histological assessment, samples from 6 fish were stained with hematoxylin and eosin (H&E), while samples from an additional 6 fish were stained with Periodic Acid-Schiff (PAS), following the previously published laboratory protocols^49,50^. PAS staining was specifically selected to evaluate the secretory activity, distribution and density of goblet cells of the intestinal mucosa of the treated fish.

### Hepatic and intestinal oxidative stress biomarkers

Clear supernatants, prepared from liver and intestinal tissue homogenates (processed in cold 0.86% saline (1:9, w/v) and centrifuged (13,600 × g, 10 min, 4 °C), were used for evaluation of the oxidative stress biomarkers. Liver antioxidant analyses including: total antioxidant capacity (T-AOC) (according to FRAP assay, 520 nm; Benzie and Strain^51^), MDA concentrations (according to TBARS assay, 532 nm; Ohkawa, et al.^52^), and GST enzyme activity (340 nm; Habig, et al.^53^). Intestinal analyses measured: SOD (560 nm; Marklund and Marklund^54^), CAT (240 nm; Aebi^55^), and GPx activity (340 nm; Paglia and Valentine^56^). Commercially available kits from Cusabio Biotech Company, Ltd. (Wuhan, China) and kits from Biodiagnostic Co. (Giza, Egypt) were used for these evaluations.

### RT-qPCR workflow

Following RNA extraction from three intestinal samples from each experimental group (n = 3) with TRIzol reagent (Invitrogen, USA), cDNA was synthesized using a High-Capacity cDNA Reverse Transcription Kit after confirming RNA integrity and concentration with a NanoDrop (Applied Biosystems, USA). All samples were stored at -80 °C. The total RNA purity was assessed using spectrophotometry; all samples used in this study exhibited an A260/A280 ratio between 1.8 and 2.1, indicating high-quality RNA free of protein contamination. The integrity was further confirmed via agarose gel electrophoresis, showing distinct 28S and 18S ribosomal RNA bands with no visible genomic DNA contamination. Using qPCR with SYBR Green Master Mix (Applied Biosystems, USA), we measured the expression of growth- (*GHR1*, *GHR2*, *IGF-1*, *IGF-2*), inflammation- (*IL-1β*, *TNF-α*, *caspase 3*), and immune-related (*TLR2*, *INF-γ*) genes. Primer specifications are listed in Table 3. β-actin was selected based on its established stability under similar experimental conditions in our previously published studies. Furthermore, the Ct values for β-actin remained constant across all experimental groups (P > 0.05), confirming its suitability as an internal control for this specific model. For quantification, the gene expression was quantified using the 2^−ΔΔCT^ method according to the protocols described in detail by Livak and Schmittgen^57^. Results are presented as fold-change relative to the control group, with data expressed as mean ± SE.

**Table 3:** Primers used for relative quantitative real-time PCR.

Target mRNA	Primer name	Primer	Primer sequence (5′–3′)	NCBI GenBank Accession No	Published in
Housekeeping gene					
*β-actin*	β-actin-F	Forward	ATCGTGGGGCGCCCCAGG	EU887951.1	(Chaklader et al. 2024)
	β-actin-R	Reverse	CTCCTTAATGTCACGCACGATTTC		
Immune-related genes					
*TLR2*	TLR-2-F	Forward	AAAAGCATAGATGAGTTCCACATCC	JQ809459.1	(Jia et al. 2019)
	TLR-2-R	Reverse	GTAAGACAAGGCATCACAAACACC		
*INF-γ*	INF-γ-F	Forward	GGGTGGTGTTTTGGAGTCGT	NM_001287402.1	(Hendam et al. 2024)
	INF-γ-R	Reverse	CATCTGTGCCTGGTAGCGAG		
Growth-related genes					
* GHR1*	GHR1-F	Forward	TCTCAGCAGAACCGATTAATGA	AY973232.1	(Hendam et al. 2024)
	GHR1-R	Reverse	TTTGATTTTGGGTGCAGGA		
* IGF-1*	IGF-1-F	Forward	CATCGTGGACGAGTGCTG	EU272149.1	(Hendam et al. 2024)
	IGF-1-R	Reverse	ACAGGTGCACAGTACATCTCAAG		
* IGF-2*	IGF-2-F	Forward	CGCTGCAGTTTGTCTGTGA	EU272150.1	(Hendam et al. 2024)
	IGF-2-R	Reverse	GTCGGTTGTTACCCCTGCT		
* GHR2*	GHR2-F	Forward	GAAGTTCAGCACCGAGACAA	EF052862.1	(Hendam et al. 2024)
	GHR2-R	Reverse	ACTCGAAACAGGGTCAACCA		
Inflammation-related genes					
* IL-1β*	IL-1β-F	Forward	TGCACTGTCACTGACAGCCAA	DQ061114.1	(Hendam et al. 2024)
	IL-1β-R	Reverse	ATGTTCAGGTGCACTATGCGG		
* TNF-α*	TNF-α-F	Forward	CCAGAAGCACTAAAGGCGAAGA	AY428948.1	(Abdel-Latif et al. 2022)
	TNF-α-R	Reverse	CCTTGGCTTTGCTGCTGATC		
* Caspase 3*	Caspase 3-F	Forward	GGCTCTTCGTCTGCTTCTGT	GQ421464.1	(Abdel-Latif et al. 2022)
	Caspase 3-R	Reverse	GGGAAATCGAGGCGGTATCT		

β-actin, Beta-actin; TLR2, toll-like receptor 2; GHR1, growth hormone receptor 1; GHR2, growth hormone receptor 2; IGF-1, insulin-like growth factor 1; IGF-2, insulin-like growth factor 2; TNF-α, tumor necrosis factor alpha; IL-1β, interleukin 1 beta; INF-γ, interferon gamma.

### Statistical analysis

All data were expressed as mean ± SE and tested for normality (via the Shapiro–Wilk test) and homogeneity of variances (via Levene’s test). Differences among dietary treatments were analyzed using ANOVA followed by Dunnett’s multiple comparison test to identify significant differences. Values denoted by different letters were considered statistically significant at *p* < 0.05.

## Results

### Growth performance, feed utilization, and survival

Table 4 Initial body weights were comparable across all groups (*p* = 0.81). Final body weight, weight gain, feed intake, feed conversion ratio (FCR), and specific growth rate (SGR) were significantly affected by dietary treatments. Fish fed the high soybean meal (SB) diet showed the poorest performance, with significantly lower final body weight (55.70 ± 0.8 g) and weight gain (36.23 ± 0.4 g) compared to the CNT (*p* = 0.0002 and *p* = 0.0001, respectively). Supplementation with sodium butyrate (SB + BT), sodium propionate (SB + PR), or their mixture (SB + PR + BT) significantly improved growth parameters relative to SB-fed fish. The mixture diet yielded the highest final body weight (71.00 ± 1.5 g), weight gain (51.20 ± 1.4 g), and SGR (1.42 ± 0.0%), while FCR was significantly reduced in supplemented groups (*p* = 0.0001), indicating enhanced feed efficiency. Feed intake was significantly elevated in the SB and SB + PR + BT groups compared to others (*p* = 0.0001). Survival remained 100% across all treatments.

**Table 4:** Effects of dietary sodium propionate (PR), sodium butyrate (BT), and their mixture (PR + BT) on the growth performance parameters and survival of Nile tilapia fed high soya bean meal (SB) diet for a period of 12 weeks.

Performance parameters	Experimental groups					*P* values
	CNT	SB	SB + BT	SB + PR	SB + PR + BT	
Initial body weight (g)	19.63 ± 0.1	19.48 ± 0.4	19.85 ± 0.4	19.50 ± 0.1	19.80 ± 0.1	0.81
Final body weight (g)	59.22 ± 0.6^c^	55.70 ± 0.8^d^	68.31 ± 0.4^b^	69.28 ± 0.7^b^	71.00 ± 1.5^a^	0.0002
Feed intake (g/fish)	65.63 ± 0.1^b^	74.88 ± 0.0^a^	65.55 ± 0.9^b^	65.93 ± 0.1^b^	74.20 ± 0.7^a^	0.0001
Weight gain (g)	39.59 ± 0.1^c^	36.23 ± 0.4^d^	48.46 ± 0.1^b^	49.78 ± 0.6^a^	51.20 ± 1.4^a^	0.0001
Feed conversion ratio	1.66 ± 0.0^b^	2.07 ± 0.0^a^	1.35 ± 0.0^c^	1.32 ± 0.0^c^	1.45 ± 0.0^c^	0.0001
Specific growth rate (%/day)	1.23 ± 0.0^b^	1.17 ± 0.0^c^	1.37 ± 0.0^a^	1.41 ± 0.0^a^	1.42 ± 0.0^a^	0.0003
Survival rate (%)	100 ± 0.0	100 ± 0.0	100 ± 0.0	100 ± 0.0	100 ± 0.0	–

Data is presented as means ± SE (n = 3).

Values with different superscript letters in the same row are considered as statistically significantly different within groups (*P* < 0.05).

### Digestive enzyme activities

Dietary supplementation had a significant influence on intestinal digestive enzymes (all *p* < 0.0001) (Table 5). Lipase activity was significantly reduced in the SB group compared with the CNT group, but was restored in the SB + BT, SB + PR, and SB + PR + BT groups, with values in the mixture and CNT groups being statistically comparable. Amylase and protease activities showed similar patterns, where SB-fed fish exhibited significantly lower enzyme activities (*p* < 0.0001), while supplementation with propionate, butyrate, or their mixture enhanced enzyme activities, with the highest levels observed in SB + PR + BT approaching CNT values.

**Table 5:** Effects of dietary sodium propionate (PR), sodium butyrate (BT), and their mixture (PR + BT) on the intestinal digestive enzyme activities of Nile tilapia fed high soya bean meal (SB) diet for a period of 12 weeks.

Digestive enzymes	Experimental groups					*P* values
	CNT	SB	SB + BT	SB + PR	SB + PR + BT	
Lipase activity (U/ mg protein)	9.75 ± 0.16^a^	5.22 ± 0.36^c^	7.71 ± 0.13^b^	7.74 ± 0.20^b^	9.07 ± 0.25^a^	< 0.0001
Amylase activity (U/mg protein)	7.82 ± 0.13^a^	4.19 ± 0.29^d^	6.19 ± 0.10^c^	6.21 ± 0.16^bc^	7.18 ± 0.30^ab^	< 0.0001
Protease activity (U/mg protein)	5.77 ± 0.09^a^	3.09 ± 0.21^d^	4.56 ± 0.08^c^	4.58 ± 0.12^bc^	5.30 ± 0.22^ab^	< 0.0001

Data is presented as means ± SE (n = 3).

Values with different superscript letters in the same row are considered as statistically significantly different within groups (*P* < 0.05).

### Intestinal histomorphology

**Fig. 1:**
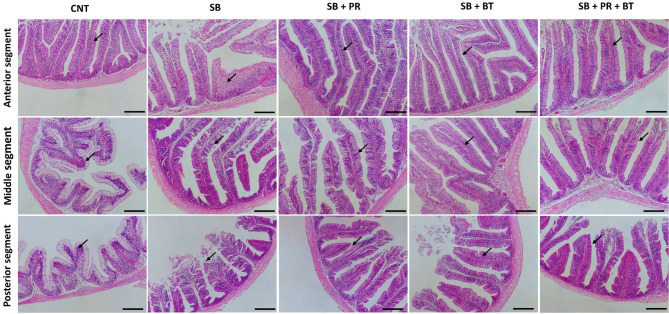
Representative histophotomicrographs including the anterior part (upper panel), middle part (middle panel) and posterior part (lower panel) of the Nile tilapia intestine in the control (CNT) group, a group fed high soybean meal (SB), SB diet supplied with sodium propionate (PR), SB diet supplied with sodium butyrate (BT), and SB diet supplied with a mixture of propionate and butyrate (PR + BT). The histological structure in all groups except SB group showed normal, intact intestinal villi (V) and intestinal wall. SB group showed signs of enteritis through all segments with erosion of intestinal villi and inflammatory cells infiltration. The best histological appearance of the intestinal section to alleviate the negative effects of SB effects appeared in the mixture group followed by BT then PR groups. Stain H&E. Bar: 100 µm.

**Fig. 2:**
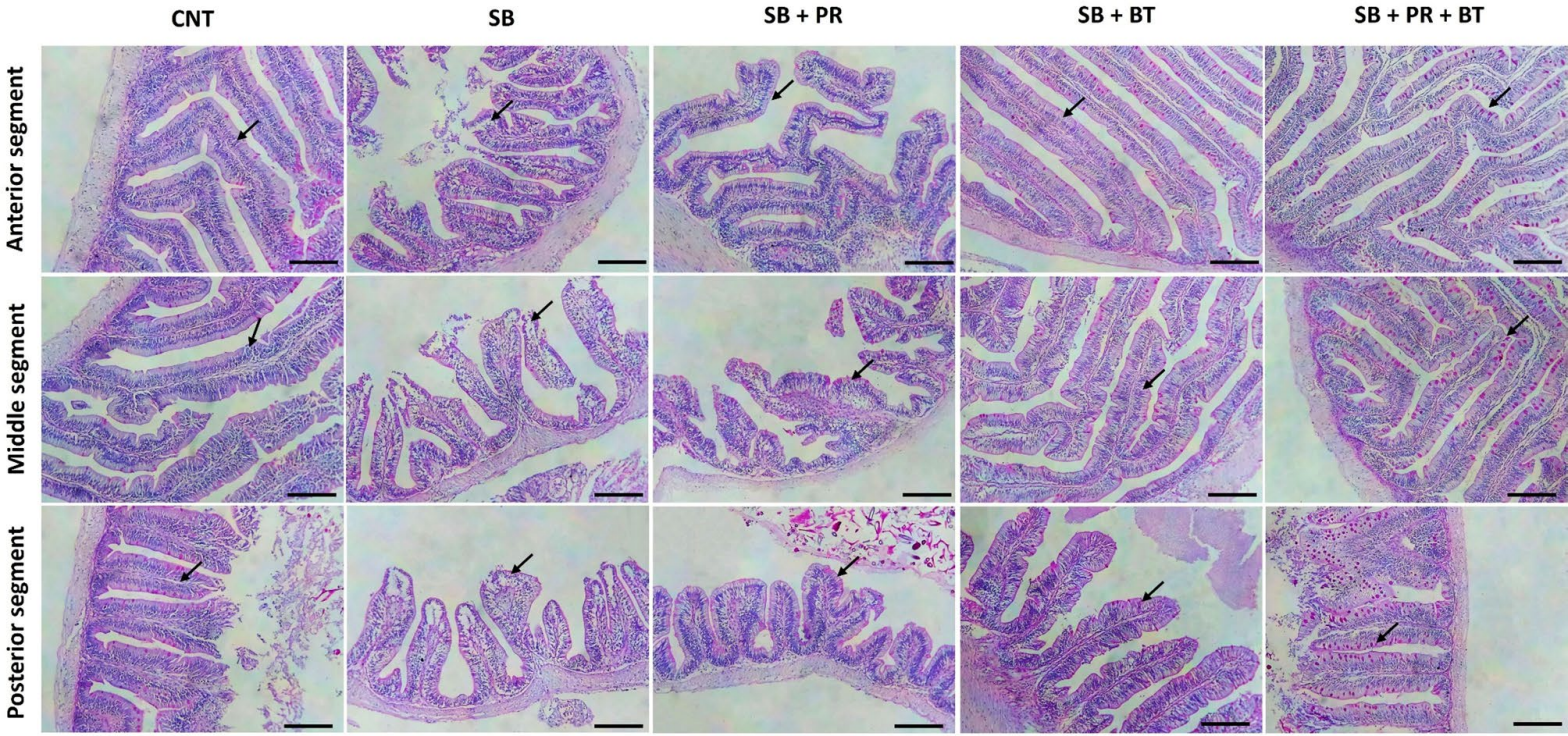
Representative histophotomicrographs including the anterior part (upper panel), middle part (middle panel) and posterior part (lower panel) of the Nile tilapia intestine in the control (CNT) group, a group fed high soybean meal (SB), SB diet supplied with sodium propionate (PR), SB diet supplied with sodium butyrate (BT), and SB diet supplied with a mixture of propionate and butyrate (PR + BT). The histological structure in all groups except SB showed normal, intact intestinal villi (V) and intestinal wall. SB group revealed signs of intestinal degeneration and sloughing of the intestinal villi. There was a clear improvement in the histological appearance with abundant goblet cells particularly in the mixture group as well as the BT group. Stain PAS. Bar: 100 µm.

The histological examination of intestinal sections revealed a standard morphology in the control group, with all layers of the intestinal wall (mucosa, propria, submucosa, muscularis, and serosa) presenting a typical, undisturbed appearance. The villi and associated crypts were intact, and the enterocytes were arranged in an orderly manner (Figs. 1, 2). Conversely, the group fed a high soybean meal diet (SB) displayed clear signs of enteritis across all intestinal segments, including villus erosion and infiltration by inflammatory cells. The architectural integrity of the intestinal villi was markedly improved in groups where the SB diet was supplemented with propionate (PR), butyrate (BT), or a mixture of both (PR + BT) (Fig. 1). An additional finding was a higher abundance of PAS-stained goblet cells interspersed among the enterocytes in the PR, BT, and PR + BT groups, with the greatest density observed in the combination treatment group (Fig. 2).

### Oxidative stress biomarkers

Oxidative stress markers were significantly altered by dietary treatments (Table 6). In the liver, T-AOC was reduced in the SB group compared with CNT but significantly restored in supplemented groups (*p* = 0.004). MDA levels were elevated in SB-fed fish but significantly reduced by supplementation, with the lowest values observed in SB + PR + BT (*p* < 0.0001). GST activity was suppressed in SB but significantly enhanced in SB + BT and SB + PR + BT groups (*p* = 0.003). In the intestine, SOD, CAT, and GPx activities were markedly depressed in the SB group but were significantly increased in supplemented groups (all *p* ≤ 0.001), with SB + PR + BT restoring activities closest to CNT levels.

**Table 6:** Effects of dietary sodium propionate (PR), sodium butyrate (BT), and their mixture (PR + BT) on the oxidative stress biomarkers of liver and intestines of Nile tilapia fed high soya bean meal (SB) diet for a period of 12 weeks.

Oxidative stress biomarkers	Experimental groups					*P* values
	CNT	SB	SB + BT	SB + PR	SB + PR + BT	
Liver						
T-AOC (U/mg protein)	24.17 ± 0.69^a^	15.99 ± 2.08^b^	22.14 ± 0.6^ab^	23.41 ± 1.25^a^	26.53 ± 1.83^a^	0.004
MDA (nmol/mg protein)	25.10 ± 0.23^a^	28.87 ± 0.36^a^	24.06 ± 0.82^bc^	24.92 ± 0.33^b^	22.25 ± 0.57^c^	< 0.0001
GST (U/mg protein)	3.50 ± 0.50^ab^	1.22 ± 0.22^b^	4.25 ± 0.89^a^	3.66 ± 0.30^ab^	5.62 ± 0.59^a^	0.003
Intestine						
SOD (U/mg protein)	3.47 ± 0.10^a^	1.10 ± 0.03 ^b^	3.04 ± 0.44^a^	2.77 ± 0.40^a^	3.40 ± 0.26^a^	0.001
CAT (U/mg protein)	7.69 ± 0.22^a^	2.44 ± 0.08 ^b^	7.61 ± 0.13^a^	7.61 ± 0.16^a^	8.03 ± 0.52^a^	< 0.0001
GPx (U/mg protein)	52.68 ± 1.49^a^	16.69 ± 0.51^d^	43.24 ± 0.63^c^	48.44 ± 0.51^b^	50.92 ± 0.34^ab^	< 0.0001

Data is presented as means ± SE (n = 3).

Values with different superscript letters in the same row are considered as statistically significantly different within groups (*P* < 0.05).

T-AOC, total antioxidant capacity; MDA, malondialdehyde; GST, glutathione-S-transferase; SOD, superoxide dismutase; CAT, catalase; GPx, glutathione peroxidase.

### Gene expression profiles

**Fig. 3:**
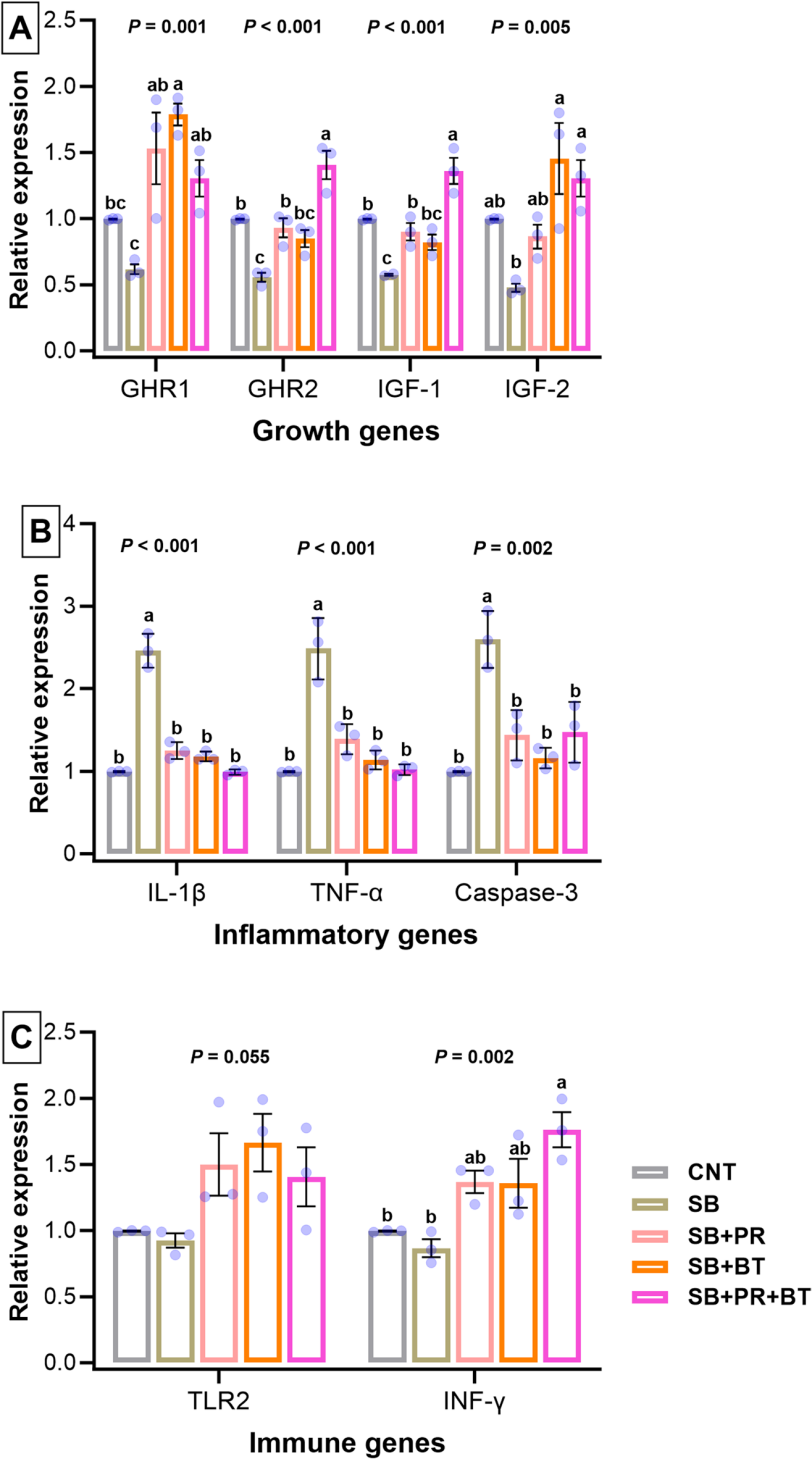
Effects of dietary sodium propionate (PR), sodium butyrate (BT), and their mixture (PR + BT) on the relative expression of (**A**) growth-related (*GHR1*, *GHR2*, *IGF-1*, and *IGF-2*), (**B**) inflammation-related (*IL-1β*, *TNF-α*, and *caspase 3*), and (**C**) immune-related (*TLR2* and I*NF-γ*) genes in the intestines of Nile tilapia fed high soya bean meal (**SB**) diet for a period of 3 months. Data were expressed as means ± SE (n = 3). The comparison between the test groups and the control (CNT) group was carried out by one-way ANOVA analysis followed by Dunnett’s multiple comparison test. Values with different letters are considered as statistically significantly different within groups.

The expression of intestinal genes was significantly modulated by dietary treatments (Fig. 3). Growth-related genes (*GHR1*, *GHR2*, *IGF-1*, and *IGF-2*) were downregulated in the SB group but significantly upregulated in SB + PR, SB + BT, and SB + PR + BT groups, with the mixture group showing the highest expression (p < 0.05). Inflammation-related genes (*IL-1β*, *TNF-α*, and *caspase 3*) were significantly upregulated in SB-fed fish, indicating intestinal inflammation, but their expression was significantly reduced in supplemented groups, particularly SB + PR + BT (*p* < 0.05). Similarly, immune-related genes (*TLR2* and *IFN-γ*) showed significant improvement in expression in supplemented groups compared with SB (*p* < 0.05), again with the mixture group demonstrating the most pronounced effect.

## Discussion

Soybean meal (SB) is a common alternative to fishmeal in aquafeeds, but its high inclusion often causes growth suppression, intestinal inflammation, and oxidative stress due to anti-nutritional factors^58,59^. Short-chain fatty acids such as butyrate and propionate have been reported to alleviate these effects by improving digestion, gut health, antioxidant capacity, and immune regulation^60–62^. Results of the present study indicate that dietary inclusion of high SB levels adversely affected growth and feed efficiency. This was reflected in statistically significant reductions in FBW, WG, and SGR, coupled with a significant increase in the FCR. These findings are consistent with earlier reports in Chinese sucker^63^, largemouth bass^64^, tilapia, and sturgeon^65^, where excessive inclusion of SB suppressed growth and feed efficiency due to the presence of anti-nutritional factors such as β-conglycinin, glycinin, and trypsin inhibitors, which compromise feed palatability, reduce protein digestibility, and impair nutrient assimilation^66,67^. Interestingly, in the present study, survival remained unaffected, suggesting that the effects of SB inclusion were sublethal but sufficient to disrupt normal physiological functioning^64,68,69^.

Dietary BT, PR, or their combination significantly alleviated high SB negative impacts, with the mixture producing the most pronounced enhancements in growth and feed efficiency. Comparable restorative effects of BT on growth have been reported in orange-spotted grouper (*Lates calcarifer*)^70^ and golden pompano (*Trachinotus ovatus*)^71^, where butyrate supplementation enhanced body weight and reduced FCR. Similarly, PR has been shown to improve feed conversion and nutrient retention in rainbow trout (*Oncorhynchus mykiss*)^72^ and beluga sturgeon (*Huso huso*)^73^. The superior performance of the combined supplementation in the current study suggests synergistic or additive effects, which may result from their complementary roles in modulating gut health, nutrient metabolism, and hormonal regulation^74^.

The improvements in growth and feed utilization were supported by enhanced digestive enzyme activities^75^. Fish receiving the SB diet alone showed significantly depressed activities of lipase, amylase, and protease, which is consistent with earlier findings in Chinese sucker, yellow catfish, and turbot, where SB meal inclusion reduced pancreatic and brush-border enzyme activity^63,76,77^. Restoration of enzyme activity by BT and PR supplementation agrees with studies in seabass^78,79^, yellow catfish^80^, and pompano^71^, where short-chain fatty acids (SCFAs) stimulated digestive enzyme secretion and improved nutrient assimilation. The mixture group restored the enzyme activities to levels similar to the control indicating that SCFAs directly or indirectly enhance enzymatic function, possibly by promoting intestinal epithelial health and microbial fermentation processes that stimulate digestive physiology^81,82^.

Histological observations further supported these functional outcomes^83,84^. Severe enteritis, villus erosion, and inflammatory cell infiltration were evident in fish fed the SB diet, a hallmark of SB meal-induced enteropathy also described in Atlantic salmon^85^, grouper^86^, and turbot^87^. Conversely, supplementation with BT, PR, or their mixture significantly improved intestinal morphology, with more intact villi and abundant PAS-positive goblet cells, particularly in the mixture group. Similar restorative effects of BT have been reported in grouper^70^, and European sea bass^88^, where villus height, goblet cell density, and mucosal integrity were enhanced following supplementation^83^. Goblet cell proliferation plays an important role in mucosal protection through mucus secretion, which reinforces barrier function and prevents microbial translocation^89^. The current findings thus suggest that SCFAs improve intestinal architecture both structurally and functionally, contributing to enhanced nutrient absorption and resilience against inflammation.

The protective effects of BT and PR were also evident in oxidative stress biomarkers^90^. SB alone increased hepatic MDA concentrations and suppressed antioxidant enzymes such as SOD, CAT, and GPx, reflecting elevated oxidative stress. These alterations mirror findings in largemouth bass^64^ and common carp^91,92^, where SB meal–based diets compromised antioxidant defense and increased lipid peroxidation. In the present study, supplementation with SCFAs significantly restored antioxidant enzyme activities and reduced MDA levels, with the mixture achieving the closest values to the control. Comparable enhancements in antioxidant status by butyrate have been observed in turbot^39^ and tilapia^93^, where butyrate promoted redox balance and mitigated oxidative injury^22^. These findings suggest that SCFAs not only improve antioxidant enzyme capacity but also reduce reactive oxygen species production, thereby alleviating cellular damage and maintaining intestinal and hepatic homeostasis.

Gene expression analysis in this study provided molecular evidence supporting the observed physiological responses^94^. The intestine plays a central role not only in digestion and nutrient absorption but also in regulating immune function and overall health^32,95^. To date, no studies have specifically examined whether dietary supplementation with PR, BT, or their combination (PR + BT) can alleviate the negative effects of high SB inclusion on intestinal growth, modulate immune-related markers, and suppress inflammatory gene expressions in fish. Growth-related genes (*GHR1*, *GHR2*, *IGF-1*, and *IGF-2*) were markedly downregulated in the SB group, consistent with the impaired growth performance. Similar repression of the GH/IGF axis under high soybean meal conditions has been reported in turbot and Nile tilapia^96,97^. Supplementation with BT and PR reversed these trends, with the mixture showing the highest upregulation, in agreement with studies demonstrating that SCFAs stimulate the GH/IGF pathway and promote protein synthesis^98,99^.

Inflammatory markers (*IL-1β*, *TNF-α*, and *caspase-3*) were significantly upregulated in the SB group, reflecting intestinal inflammation and apoptosis, while SCFAs supplementation, particularly the mixture, suppressed their expression. These findings are consistent with earlier reports in tilapia and grouper, where BT attenuated NF-κB activation and reduced pro-inflammatory cytokines^100,101^. A research study reported that the combination of BT and PR significantly upregulates the expression of key immune genes, including *TNF-α*, *IL-1β*, *IL-10*, and *lysozyme*, in the head kidney and mucosal tissues of goldfish (*Carassius auratus*). This synergistic treatment enhances the fish’s innate immune response and increases resistance to pathogenic challenges compared to using either additive alone^22^. In another research, Mirghaed, et al.^102^ reported that BT alone upregulated *TNF-α*, *IL-1β*, and *LYZ* expression in rainbow trout. In common carp, PR supplementation alone elevated *TNF-α*, *IL-1β*, and *LYZ* gene expression^25^, and comparable results were observed in goldfish, where dietary PR (0.25–0.5%) upregulated *LYZ*, *TNF-α*, and *IL-1β* expression^103^. At the same time, immune-related genes such as *TLR2* and *IFN-γ* were enhanced in supplemented groups, suggesting improved innate immune capacity, in line with the immunostimulatory roles of SCFAs reported in seabass and carp^104,105^. Taken together, this study demonstrates that BT and PR alleviate the negative effects of high SBM inclusion through improvements in digestion, intestinal integrity, antioxidant defense, and regulation of growth- and immunity-related genes. The superior outcomes observed with their combined use indicate complementary modes of action, underscoring the potential of SCFAs as functional additives to counteract plant protein–induced challenges in aquaculture nutrition.

## Conclusion and perspectives

While high-soybean (SB) meal diets typically compromise growth, intestinal health, and oxidative balance in Nile tilapia, supplementation with organic acid salts (OAs) like sodium propionate (PR), sodium butyrate (BT), or their combination (PR + BT) effectively counteracted these stressors. The synergistic combination of both additives provides the most robust improvements in feed efficiency, antioxidant capacity, and gut integrity, positioning them as essential functional tools for optimizing plant-based aquafeeds. Moving forward, these benefits should be validated under long-term commercial conditions and across diverse species to further enhance the sustainability of global aquaculture.

## Data Availability

The data set is available from the corresponding authors upon reasonable request.
